# Effects of a scoring aid on glasgow coma score assessment and physicians’ comprehension: a simulator-based randomized clinical trial

**DOI:** 10.1007/s00415-024-12825-z

**Published:** 2024-12-12

**Authors:** Paulina S. C. Kliem, Kai Tisljar, Pascale Grzonka, Sebastian Berger, Simon A. Amacher, Gian Marco De Marchis, Tolga D. Dittrich, Sabina Hunziker, Stephan Rüegg, Stefano Bassetti, Roland Bingisser, Stephan Marsch, Raoul Sutter

**Affiliations:** 1 Clinic for Intensive Care Medicine, University Hospital Basel, Basel, Switzerland; 2 Department of Neurology and Stroke Center, Cantonal Hospital St. Gallen, St. Gallen, Switzerland; 3 Medical Faculty of the University of Basel, Basel, Switzerland; 4 Department of Neurology, University Hospital Basel, Basel, Switzerland; 5 Medical Communication and Psychosomatic Medicine, University Hospital Basel, Basel, Switzerland; 6 Division of Internal Medicine, University Hospital Basel, Basel, Switzerland; 7 Department of Emergency Medicine, University Hospital Basel, Basel, Switzerland

**Keywords:** Glasgow Coma Score, Randomized controlled trial, Intensive care, Neurocritical care

## Abstract

**Purpose:**

Examining the impact of scoring aids on the accuracy of assessing the Glasgow Coma Score (GCS) in a standardized trauma scenario (primary outcome). Evaluating physicians’ understanding of the GCS assessment and clinical application (secondary outcome).

**Materials and methods:**

This randomized trial was performed at the simulator center of a Swiss tertiary academic medical hospital. Participants included intensivists, emergency physicians, internists, and neurologists. The setting involved a trauma patient portraying a GCS of 8 (eyes 1, verbal 2, motor 5). Participants were randomized to receiving or not receiving a scoring aid. Video/audio recordings of the assessments and questionnaires were analyzed by two investigators.

**Results:**

Among 109 participants, 55 received a scoring aid. Overall, 52% scored correctly (score interquartile range 7–8); 43% scored too low and 90% scored within a range of ± 1. A scoring aid increased accuracy (62% vs. 43%, *p* = 0.045) and participants’ confidence, whilst decreasing assessment duration. Clinical experience further improved reliability. 89% found assessing a GCS of 8 most challenging, particularly with motor response evaluation (64%). 26% indicated tracheal intubation to be mandatory with a score of GCS ≤ 8.

**Conclusions:**

GCS assessment is improved by professional experience and a scoring aid, the use of which needs to be promoted in daily clinical practice. Frequent inaccuracy and misunderstanding regarding clinical applications may alter patient management and misguide treatment and prognosis.

**Trial registration:**

ISRCTN registry (IDISRCTN12257237) https://www.isrctn.com/ISRCTN12257237 Retrospectively registered (last amendment 08/22/2023).

**Supplementary Information:**

The online version contains supplementary material available at 10.1007/s00415-024-12825-z.

## Introduction

Since its introduction in 1974 [1], the Glasgow Coma Scale (GCS) is commonly used worldwide to describe the level of consciousness [2] and to predict outcomes [3–7] of a wide variety of critically ill medical and trauma patients. The GCS has diverse applications beyond clinical practice, including participant’s trial eligibility determination, baseline severity adjustments for clinical research, and severity scores for categorizing cohorts in intensive care units (ICUs). Notable examples of such scores are the Sequential Organ Failure Assessment (SOFA) score, the Acute Physiology And Chronic Health Evaluation (APACHE) score, the Simplified Acute Physiology Score (SAPS) and their subsequent versions [8–10]. A near-linear correlation has been observed between decreasing overall sum scores and increasing mortality in patients with traumatic brain injury [11]. The motor component of the GCS has been identified as a robust predictor of unfavorable outcomes in cases of moderate to severe brain trauma [12]. Despite its universal use, concerns about the reliability and inter-rater variability of the GCS persist. Although some studies suggest higher reliability of the GCS components compared to the sum score [13], other investigations have identified various factors influencing its reliability, such as education, training, level of consciousness, and underlying pathology [14]. Most of these few studies are limited in terms of heterogeneity in the type of reliability estimates used, patient cohorts [15], simulated scenarios [16], or video recordings [17, 18], unstandardized clinical settings and observers’ characteristics [13]. Only few attempts have been made to explore interventions aimed at enhancing scoring reliability. Previous investigations used specific clinical scenarios presented only in written form, encompassing different GCS ranges [16] instead of centering on a specific GCS with clinical implications. Examining the quality of a specific GCS assessment and measures for potential optimization are lacking, but have potential clinical implications, as for example a GCS of 8 or less in trauma patients defines severe traumatic brain injury requiring specific treatment algorithms including advanced airway management [19]. Investigating the evaluation of a particular GCS in clinical studies faces constraints due to the scarcity of patients displaying a specific GCS and limited training time for physicians to assess such cases, considering the potential delay of critical examinations and treatments. A high-fidelity simulator offers a solution by providing a standardized simulation of the targeted clinical scenario. The video and audio recordings of the scenario and the physicians’ performances allow a supervision of the simulation to assure optimal standardization and video-based structured debriefing sessions.

The aim of this study was to examine the effect of utilizing a scoring aid on the accuracy and duration of the assessment of a simulated patient with a GCS of 8 within a highly standardized clinical scenario and to evaluate the physicians’ comprehension of the GCS regarding assessment, interpretation, and clinical application.

## Material and methods

### Ethics and consent

The study was approved by the local ethics committee (EKNZRequ_2019-00168), which ascertained that this study complies with the general ethical principles for research involving human subjects (according to article 51(2) of the Human Research Act). Consent was received from the volunteering physicians. The Reporting Guidelines for Health Care Simulation Research—and extensions to the CONSORT (CONsolidated Standards Of Reporting Trials) and STROBE (Strengthening The Reporting of OBservational studies in Epidemiology) Statements—for randomized trials were followed. The study was registered online at the digital SRCTN registry (ID ISRCTN12257237) [20].

### Setting and study design

This investigator-initiated randomized clinical simulator-based trial was performed from 2019 to 2023 at the simulation center of the ICU at the University Hospital Basel, a Swiss academic tertiary care center.

A simulator-based emergency training was offered to resident physicians specializing in intensive care medicine, emergency medicine, internal medicine, and neurology. None of the physicians had received any specific training in GCS assessment and participants were not remunerated. Information sharing between participants was prohibited. Debriefing sessions were conducted individually after each training and the identities of participants were not shared.

All participants completed two questionnaires prior to and after the simulation. The first included questions about age, medical and clinical knowledge/experience and specialization, and stress level. The second questionnaire asked about the stress level during the simulation, the GCS assessed and the confidence about its accuracy, which exact scores and components (eye, verbal, motor response) are difficult to assess without aids, which GCS is the highest intubated patients can reach, if patients with a GCS ≤ 8 must be intubated, and how certain they are that another physician would assess the same GCS for the identical scenario (quantifications for level of stress and certainty as described in legend of Table 1**)**.

**Table 1:** Comparison of participants’ baseline and performance characteristics between participants with and without a scoring aid

Baseline and performance characteristics	Without scoring aid (*n* = 54)		With scoring aid (*n* = 55)		*p*-value
Demographics	*n*/median	%/IQR	*n*/median	%/IQR	
Age (years; median, IQR)	32	30–36	31	30–34	0.269
Female (*n*, %)	33	61.1	33	60.0	0.906
Professional characteristics					
Stated to routinely use GCS in practice (*n*, %)	39	72.2	45	81.8	0.262
Physicians’ affiliations					0.252
Intensive care (*n*, %)	15	27.8	13	23.6	
Emergency medicine (*n*, %)	12	22.2	6	10.9	
Internal medicine (*n*, %)	12	22.2	20	36.4	
Neurology (*n*, %)	15	27.8	16	29.1	
Clinical experience (years; median, IQR)	3.8	2.5–6.0	4.0	3.0–6.0	0.966
Previous simulator training (*n*, %)	26	48.2	25	45.5	0.778
Personal conditions prior to simulator training					
Working hours prior to participation (hours; median, IQR	8.3	0–10	9	0.5–10	0.465
Self-reported stress level hours prior to participation (rated from 0 to 10^a^; median, IQR)	5	4–6	5	3–6	0.625
Performance Characteristics					
GCS assessed (median, IQR)	7.5	7–8	8	7–8	0.295
Procedure of eye response assessed correctly (*n*, %)	44	81.5	48	87.3	0.405
Procedure of verbal response assessed correctly (*n*, %)	38	70.4	44	80.0	0.244
Procedure of motor response assessed correctly (*n*, %)	31	57.4	30	54.6	0.763
Number of commands directed to the patient during assessment (median, IQR)	3	2–5	2	1–5	0.876
Pain stimuli applied (median, IQR)	1	1–1	1	0–1	0.083

^a^Predefined levels of stress: 0 representing no stress to 10 representing presumed maximal stress or highest stress level

^b^Predefined levels of certainty: 0 representing presumed complete uncertainty to 10 representing presumed absolute certainty

*IQR* interquartile range, *GCS* Glasgow Coma Score

Given the heightened risk of virus transmission during the COVID-19 pandemic the study was temporarily suspended from 2019 to 2020 to be in compliance with the stringent in-hospital hygiene and safety protocols put in place to ensure the safety of both participants and staff. This decision was necessitated by the study’s design, which involved close physical interaction between the study participants undergoing their training and a healthcare professional simulating the patient.

### Intervention

**Fig. 1:**
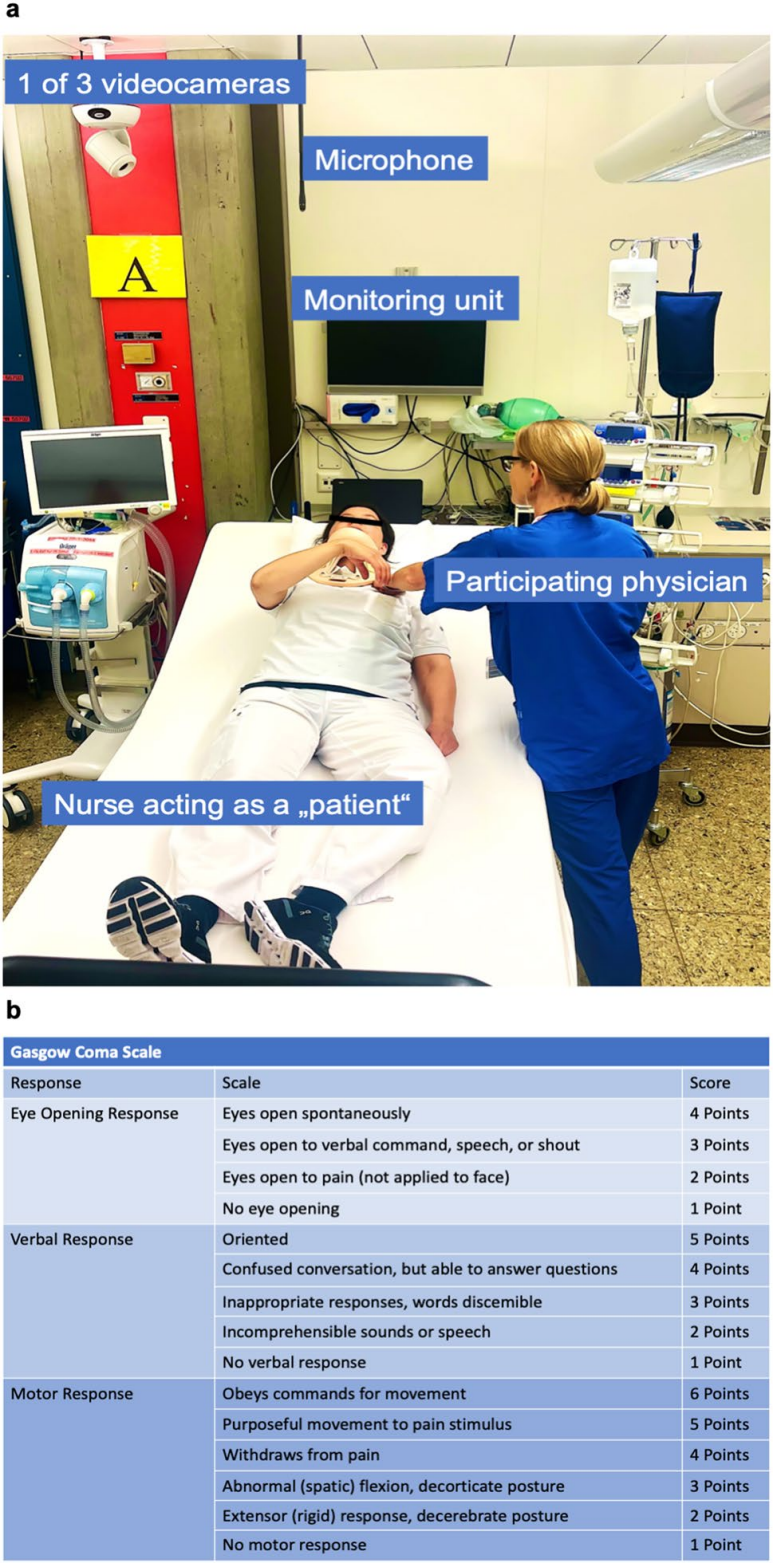
**a** The high-fidelity simulator setting; **b** the GCS scoring card used. *GCS* Glasgow Coma Score

The participants were assigned by random numbering by the principal investigator (R.S.) to either the intervention group receiving a scoring aid (i.e., GCS scoring card; Fig. 1b) or the control group.

### Simulator setup and simulated clinical scenario

Detailed information regarding the equipment of the high-fidelity simulator center was described in our prior studies[21, 22]. The scenario was video- and audio-recorded. The “simulated patient” was represented by a pretrained nurse with profound knowledge of clinical intensive care unit (ICU) management and simulator-based studies. Prior to the study, the nurse was instructed and trained to consistently present a GCS of 8 (eyes 1 point, verbal 2 points, motor 5 points). The GCS of 8 was chosen as it is the threshold to coma as well as severe traumatic brain injury requiring specific treatment algorithms [19]. Figure 1a presents the setup of the simulator, highlighting the details and components involved.

Either K.T. or R.S. confronted the participants with the adult “simulated patient” after a vehicular collision (hemodynamically stable, without airway compromise) and asked to assess the GCS. Before stopping the simulation, the participants were requested to reconfirm the assessed score.

### Data assessment

The participants’ performances were simultaneously video- and audio-recorded using frame-in-frame technology. All actions and utterances contributing to the primary and secondary endpoints were coded second-by-second and interpreted by two independent observers (P.S.C.K. & R.S). Duration of GCS assessment was defined as the time from the first contact with the “patient” to stating a score for the first time.

### Interrater variability regarding video/audio analyses

Cohen kappa (*κ*) was used to estimate interrater agreement/disagreement of the two observers regarding categorical variables and was *κ* = 0.92 for the first analyses. With interrater disagreement, the video recordings were jointly reviewed and discussed until a 100% consensus was found.

### Outcomes

The primary outcome was the accuracy of GCS assessments with or without scoring aid. Two operational definitions were employed to determine accurate GCS assessments as the primary outcome. The correct assessment of the GCS is 8, and allowing for a deviation of + −1 as a common occurrence as described by the literature [23]. The secondary outcomes were the duration of assessment with and without the scoring aid, and physicians’ knowledge regarding assessment, interpretation, and clinical applications of the GCS.

### Sample size calculation

Based on a prior study assessing GCS evaluation with and without scoring aid in multiple written scenarios presenting different levels of consciousness [16], we estimated the sample size required at 90 participants (significance level of 5% with a power of 90%).

### Statistics

Discrete variables were presented as counts with corresponding percentages; continuous variables were reported as medians with interquartile ranges (IQR). For all univariable comparisons and analyses regarding primary and secondary outcomes the Chi-square test was applied for categorical data, the Mann–Whitney test for continuous variables.

Statistical significance was determined with a threshold of ≤ 0.05. The statistical analysis was conducted using STATA®16.1 software (Stata Corp., College Station, TX, USA).

### Data availability statement

The corresponding author has access to all data. He takes full responsibility for their integrity, the accuracy of data analysis and interpretation, and the conducted research. The authors have the right to publish all data, separate from the guidance of any sponsor.

## Results

### Participants’ demographics, professional, and performance characteristics

In total, 110 simulation trainings were recorded, and 55 of the participants were supported by a scoring aid. One recording in the control group was excluded due to technical problems (supplemental Fig. 1). Participants’ baseline characteristics did not differ between the randomization groups. Demographic, professional and performance characteristics of the participants, as well as primary and secondary outcomes, are presented in the supplemental Table 1. Comparisons between participants supported by a scoring aid and those without revealed no differences in baseline and performance characteristics (Table 1).

### Primary outcomes

**Fig. 2:**
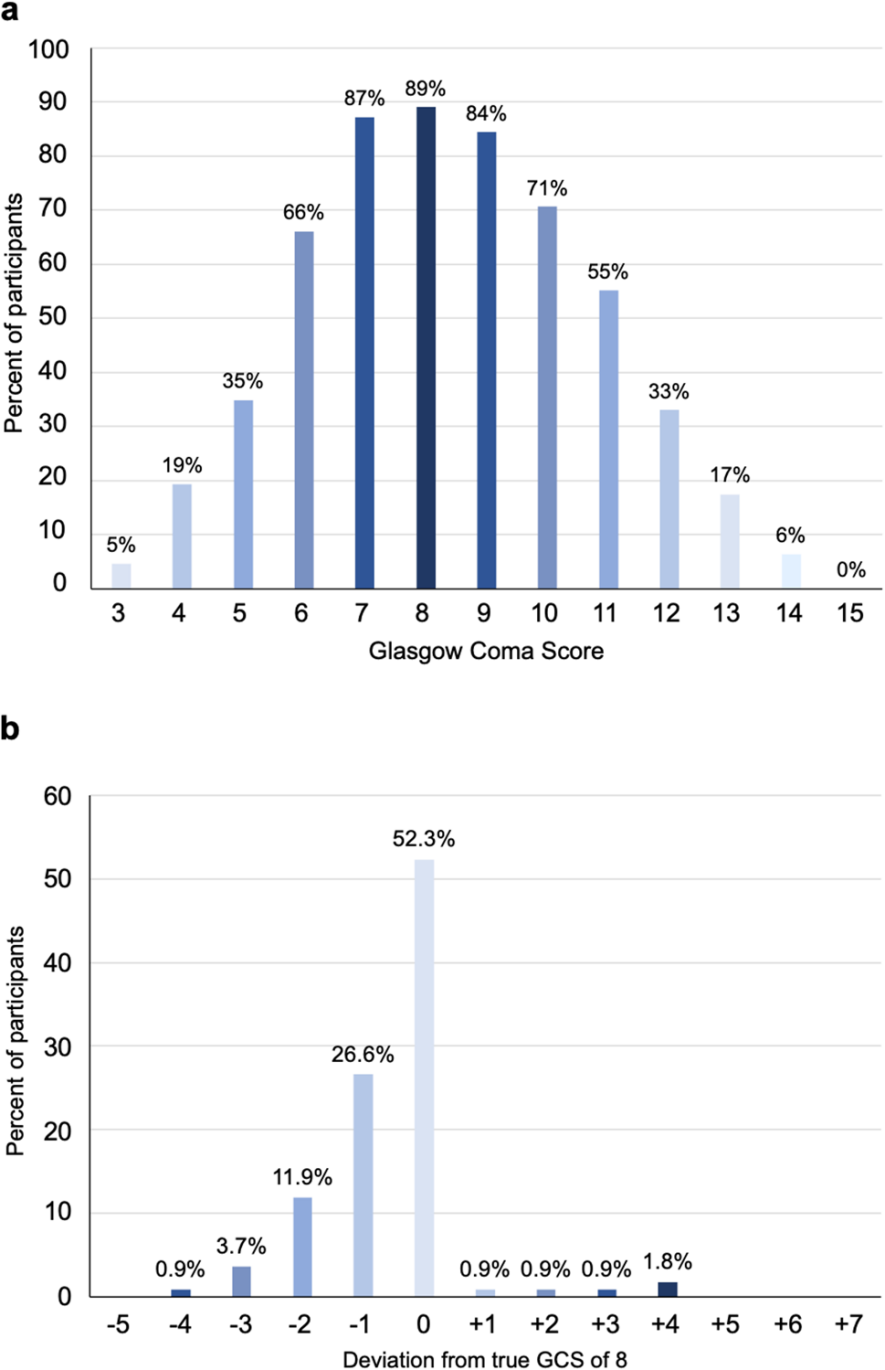
**a** Specific GCS subjectively stated to be difficult to assess without scoring (not mutually exclusive) and **b** deviation of assessed GCS from true GCS of 8 in the simulated scenario. *GCS* Glasgow Coma Score

Overall, 52% of the participants scored the correct GCS of 8 (median GCS 8, IQR 7–8; supplemental Table 1) with 43% of participants scoring the GCS too low and 5% too high (Fig. 2b). However, 90% of the participants, scored ± 1 point around the true GCS. Participants supported by a scoring aid assessed faster and more accurately (34 [61.8%] vs. 23 [42.6%], *p* = 0.045; Table 2). Within the accepted variation of ± 1 point around the true GCS, the accuracy rate improved from 70% without an assessment aid to 79% with the aid (*p* = 0.015).

**Table 2:** Comparison of primary and secondary outcomes between participants with and without a scoring aid

Outcomes	Without scoring aid (*n* = 54)		With scoring aid (*n* = 55)		*p*-value*
Primary outcome	*n*/median	%/IQR	*n*/median	%/IQR	
True GCS assessed correctly (*n*, %)	23	42.6	34	61.8	**0.045**
GCS assessment within a range of ± 1 point around the true GCS of 8 (*n*, %)	38	70.3	49	89.1	**0.015**
Secondary outcomes					
Level of certainty that assessed GCS is correct (rated from 0 to 10^b^; median, IQR)	7	5–8	8	7–9	**0.002**
Duration of GCS assessment (seconds from first touch of the patient to calling a specific score; median, IQR)	59	39–76	44	35–59	**0.024**
Understanding regarding assessment					
Checking eye response recognized as the most challenging assessment (*n*, %)	6	11.1	1	1.8	0.060
Checking verbal response recognized as the most challenging assessment (*n*, %)	18	33.3	14	25.5	0.366
Checking motor response recognized as the most challenging assessment (*n*, %)	30	55.6	40	72.7	0.061
Understanding regarding clinical applications					
Stated that patients with a GCS ≤ 8 have to be intubated (*n*, %)	16	29.6	12	21.8	0.444
Stated that patients with a GCS ≤ 8 do not have to be intubated (*n*, %)	1	1.9	3	5.5	NA
Stated that intubation should be considered depending on the clinical context in patients with a GCS ≤ 8 (*n*, %)	37	68.5	40	72.7	NA
Maximal GCS stated to be attainable in intubated patients (median, IQR)	10.5	8–11	11	10–11	0.086
Understanding regarding limitations					
Presumed interrater variability as stated by the participants (rated from 0 to 100% certainty^a^; median, IQR)	50%	40%–70%	50%	30%–70%	0.722

^a^Predefined level of presumed interrater variability: 0% representing complete uncertainty and 100% representing absolute certainty that other physicians will assess a different GCS of the same patient with an identical clinical condition

*bold font indicates statistical significance

*IQR* interquartile range, *GCS* Glasgow Coma Score

**Fig. 3:**
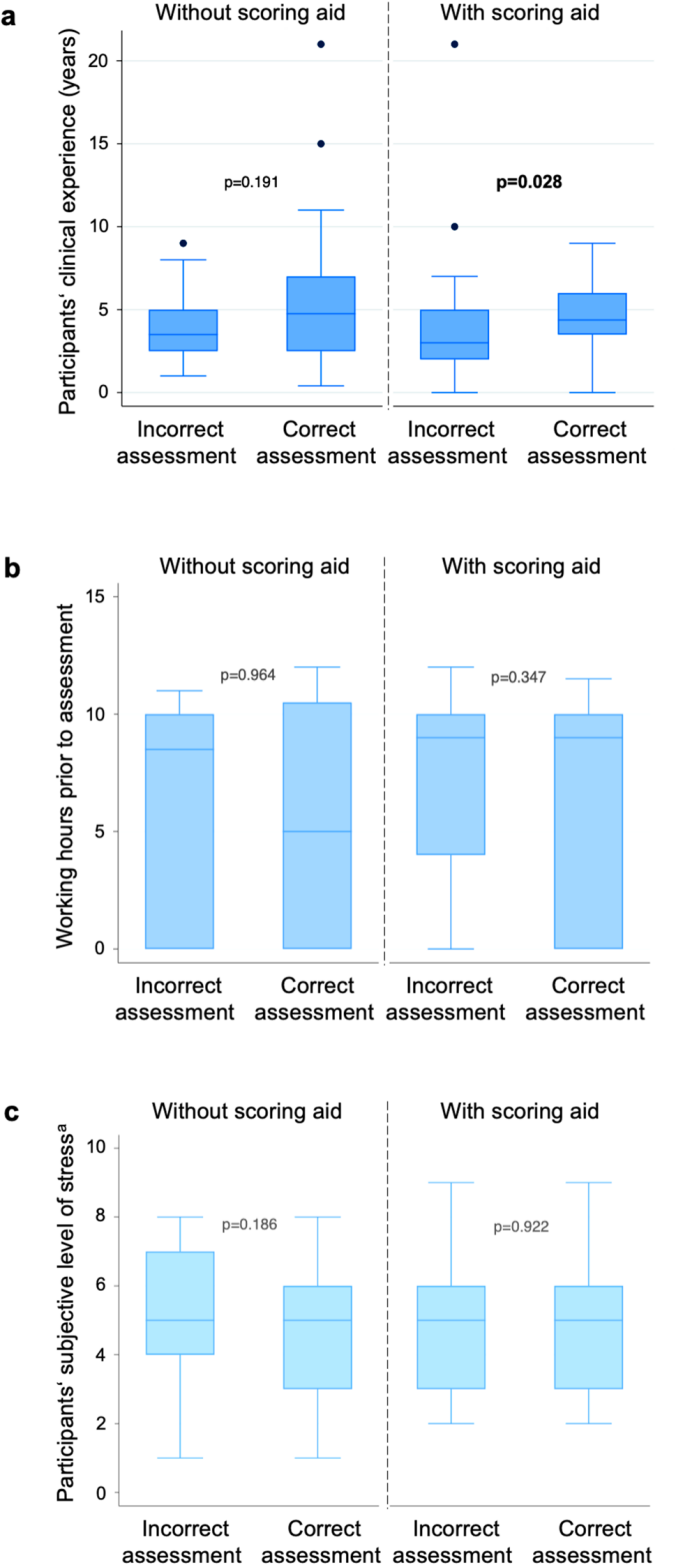
Correctness of GCS assessment (primary outcome) in dependence on participants’ clinical experience (**a**), working hours (**b**) and stress level (**c**) prior to assessment. a Predefined levels of stress: 0 representing no stress to 10 representing presumed maximal stress or highest stress level

Analyses regarding the influence of participants’ characteristics on the accuracy of the evaluated GCS are presented in Fig. 3. Only years of clinical experience revealed further improvement in assessment reliability when using the scoring aid (Fig. 3a).

Performance analyses grouped by the participants’ affiliations revealed a correctly assessed GCS in 19 of 28 (67.9%) intensivists, in 6 of 18 (33.3%) emergency medicine specialists, in 14 of 32 (43.8%) internists, and in 18 of 31 (58.1%) neurologists, overall *p* = 0.085.

### Secondary outcomes

The secondary outcomes, as assessed through the predefined questionnaires, are presented in the lower half of supplemental Table 1 as well as Table 2.

The assessment time was shorter in the intervention group. The questionnaire responses are presented in Table 2. Of the 109 participants 84 (33%) indicated not to use a scoring card in daily clinical practice (Table 2). In contrast, the participants also indicated difficulties in assessment without a scoring card (Fig. 2a) and 64% of participants struggled with the quantification of the best motor response. Accordingly, 89% of participants reported a GCS of 8 to be the most challenging. Participants in the intervention group were more confident in their accuracy compared to the controls (Table 2). The overall median level of presumed interrater agreement (predefined as a percentage range) was 50%, meaning that participants assumed a different assessment by every second colleague. Evaluating participants’ understanding regarding clinical applications, revealed a majority considering tracheal intubation to be mandatory for managing patients with a GCS ≤ 8, irrespective of the clinical context (supplemental Table 1 and Table 2).

## Discussion

This investigator-initiated simulator-based randomized clinical trial aimed to assess physicians’ knowledge of the GCS and to evaluate the impact of a scoring aid on the accuracy of GCS assessment. The randomized design, the standardized simulated clinical scenario, and the involvement of physicians from different medical specialties frequently confronted with neurological assessments are the main strengths of our study. The focus of our study on the specific GCS of 8, holding significant clinical implications for trauma management [19], and the use of a high-fidelity simulation generated by a proficiently trained nurse adds further information and advantages, compared to previous studies [16, 17]. The decision to focus our study on the GCS of 8 is further supported by our participants’ indication as being the most challenging score to assess.

Our study demonstrated that both the accuracy and the assessment time of the GCS improved when participants were supported by a scoring aid. These findings were corroborated by expressed higher levels of certainty regarding the accuracy of their assessment when using a scoring aid. In our trial, the assessment of a GCS of 8 without the assistance of a scoring aid was inaccurate in 58%. However, despite the use of a scoring aid, inaccuracy remained at 39% in the intervention group. These findings align with a previous study investigating the impact of a scoring aid on different GCS assessments of written clinical situations, reporting 75% inaccurate assessments without compared to 43% with scoring aids [16]. Additionally, in our cohort, 80% scored within a GCS range of ± 1 points, similarly to a previous study reporting an agreement between ICU staff in the range of ± 1 point in 93% of the GCS [23]. Another randomized study examining the effects of scoring aids on GCS assessments of videoed clinical scenarios observed no benefit regarding accuracy of assessment, but was limited in size and likely underpowered [17].

The present study demonstrated a statistically significant, yet limited improvement in scoring accuracy, even when considering the additional improvement by increased clinical experience. These limited effects cannot be attributed to other differences between the well-balanced groups examined.

The finding that the use of a scoring card enhances scoring accuracy may appear predictable at first glance and the information presented on the GCS reference cards is readily available online or in coat pocketbooks in every emergency room. In our study, however, 33% of participating clinicians stated not to use such a GCS reference card, a finding that mirrors the clinical experience of the authors with many clinicians and ICU nurses carrying such reference cards, but hardly using them. Some reasons may be the perception that referring to such a simple card may be time-consuming in emergency situations or the concern that reliance on such a “basic tool” for routine assessments might give an impression of inadequate proficiency or lack of confidence in their clinical abilities. Another possible factor may be the lack of strong evidence that the use of a GCS reference card improves the accuracy of the GCS assessment. Further research is needed to investigate the best possible implementation strategies taking such potential barriers to the use in daily clinical practice into account.

The scientific and clinical contribution of our study lies in its highly standardized, structured, and simulation-based approach and its focus on the application of GCS in a specific, clinically relevant scenario. Our study addresses critical gaps that prior research has either not explored or insufficiently examined, particularly regarding the following five points: (1) Unlike previous studies that often relied on varied clinical settings, unstandardized observer backgrounds [13], written or video-recorded scenarios [16–18], our research employs a high-fidelity simulator to present a specific, challenging GCS case—a trauma patient with a GCS score of 8. This controlled environment allows for more consistent assessment conditions, minimizing confounding variables that likely affect real-world GCS accuracy. The use of video and audio recordings strengthens the study by enabling detailed, consistent review—an important yet uncommon level of precision in GCS reliability research; (2) In contrast to prior studies that have tended to address broader GCS ranges, potentially diluting findings relevant to acute care, our study focused on a specific GCS of 8 highlighting a critical threshold with specific implications for trauma management (e.g., potential need for tracheal intubation), where scoring accuracy directly affects clinical decision-making; (3) By evaluating participants’ comprehension of GCS scoring and application, we identified common misconceptions that could alter patient management, as discussed further below; (4) By demonstrating that a scoring aid significantly improves GCS accuracy, confidence, and reduces assessment duration, our study supports a feasible intervention with implications for training programs, protocols, and procedures in high-stakes environments where rapid, accurate assessments are critical; (5) The high-fidelity simulator provides a replicable model to address GCS assessment challenges, particularly benefiting less experienced physicians in complex trauma cases.

Factors, such as individual stress levels and working hours prior to assessment, had no effects on the accuracy of assessment in our cohort. While the optimizing effects of training and education are well supported by earlier studies [13, 24–27], a systematic review and meta-analysis of 41 studies on factors influencing the reliability of the GCS could not link professional background and clinical experience to accuracy of assessment [14]. Our data show an additional optimizing effect with increasing years of clinical experience in the intervention group. This finding is consistent with a study showing that postgraduate year physicians in emergency departments frequently failed to correctly score best eye, verbal, and motor responses when presented with eight different consciousness levels [18]. Another study, comparing inexperienced to experienced nursing students assessing the GCS, showed the highest error rates occurring at intermediate levels of consciousness, the motor response being the most difficult to score, and the GCS assessed lower than the true score [28].

Similarly, our participants perceived the assessment of the best motor response as the most challenging in questionnaire-based self-assessments, aligning with the actual performance results.

Further evaluation of physicians’ understanding of the assessment, interpretation, and clinical application of the GCS uncovered certain misconceptions. A significant number of physicians believed tracheal intubation to be mandatory in patients with a GCS ≤ 8, regardless of the clinical context. This is a significant finding since our and other studies [15, 28] indicate that assessment in patients with a true GCS of ≤ 8 is frequently assessed as being lower. This suggests a tendency towards overly aggressive intubation in patients experiencing temporary, short-lasting alterations in consciousness with preserved protective reflexes, e.g., in postictal states or intoxications. This misconception poses significant risks, as tracheal intubation is a high-risk procedure. Approximately 45% of patients experience at least one major peri-intubation adverse event [29] (including cardiovascular instability [43%], severe hypoxemia [9%], and cardiac arrest [3%]) with an increased risk of 28-day mortality. Conversely, in a large retrospective Swiss cohort of patients with ethanol intoxication and a GCS ≤ 8, withholding intubation was not associated with severe complications [30]. Additionally, a recent systematic review found insufficient evidence supporting intubation in trauma and non-traumatic patients with a GCS ≤ 8 in the prehospital or in-hospital emergency setting [31]. Hence, a decision to intubate should never solely be based on the GCS and appropriate decision-making regarding tracheal intubation in patients with a GCS ≤ 8 should be emphasized.

Recent studies in tertiary care centers show insufficient knowledge regarding the use and application of the GCS in 10 to 50% of physicians [15, 18] and nurses [32, 33].

This corresponds to the high level of uncertainty of our participants regarding their own assessment and might have led to the presumption that every other physician would assess a different GCS in the same scenario. Such uncertainty, imprecision and impressions may be overcome with more recently developed scoring systems, such as the FOUR (Full Outline of UnResponsiveness) scale [34]. Adopting the routine use of the FOUR scale in place of the GCS may not be particularly advantageous, as studies have shown no significant benefits of using the FOUR scale over the GCS in clinical practice [35].

The FOUR scale offers a more detailed neurological assessment than the GCS by evaluating brainstem reflexes, respiratory patterns, and the ability to detect locked-in syndrome or vegetative states—areas where the GCS is limited and lead to superior performance regarding the prediction of mortality in ICU settings than the GCS [34, 36, 37]. However, studies providing evidence that the FOUR score allows more reliable quantitative assessment of altered levels of consciousness (with the exception of altered consciousness in combination with affected brainstem function) are lacking and the increased complexity of assessing the FOUR score in clinical practice may require more training and experience to apply accurately, potentially exacerbating inter-rater variability already observed with the GCS. As our study has shown incorrect scoring is frequent with the GCS, often due to inconsistent application or misunderstanding of its criteria, these same issues are likely to persist with the FOUR score, potentially even increasing due to its more intricate structure. Furthermore, the routine adoption of the FOUR score would require significant resources for training and integration into clinical practice.

### Limitations

The single-center design may limit generalizability. As most physicians, however, are trained in multiple centers during their curriculum, our results are likely to be representative of Swiss clinicians. The use of a simulator setting in this study may have certain limitations in terms of transferability and generalizability. While a specifically trained nurse is portraying the patient, the extent to which the simulator can accurately replicate real-life scenarios is inherently constrained. While our study’s focus on a specific predefined GCS of 8 is a strength, it also restricts generalizability. However, our results align with other studies that have investigated the effects of scoring aids on GCS assessments across patients with varying GCS scores. Therefore, the limitations imposed by the focus on a single GCS value are likely mitigated by the consistency of findings across studies. This pilot study allows subsequent studies to explore physicians’ performances on other specific GCS. Lastly, our study design did not allow analyses regarding the effects of underlying pathologies and the type of stimuli applied on the assessment of the GCS, as previously suggested [14].

## Conclusions

Despite some improvement in GCS assessment by scoring aids and experience, frequent inaccuracy and misunderstanding regarding clinical applications may alter patient management and misguide treatment and prognosis. These results, combined with the widespread utilization and accepted value of the GCS as a rapid clinical neurological assessment and monitoring tool, emphasize the urgent need for educational interventions and enhanced, intensive training programs. These initiatives should not only aim to improve the assessment procedures but should also further enhance the comprehension of the GCS and promote its appropriate clinical application to optimize GCS-guided treatment and prognostication of the critically ill.

## Supplementary Information

Below is the link to the electronic supplementary material.

Supplementary file1 Supplemental Figure 1. Flow chart (TIFF 915 KB)

Supplementary file2 (DOCX 18 KB)

Supplementary file3 (DOC 219 KB)

## Data Availability

The datasets used and/or analyzed during the current study are available from the corresponding author upon reasonable request. Restrictions apply to the availability of the data to guarantee the anonymity of the volunteering physicians.
