# Supplementary figures and images for: Effects of a scoring aid on glasgow coma score assessment and physicians’ comprehension: a simulator-based randomized clinical trial
Source: J Neurol. 2024 Dec 12;272(1):57. doi: 10.1007/s00415-024-12825-z (PMC11638317; doi:10.1007/s00415-024-12825-z)

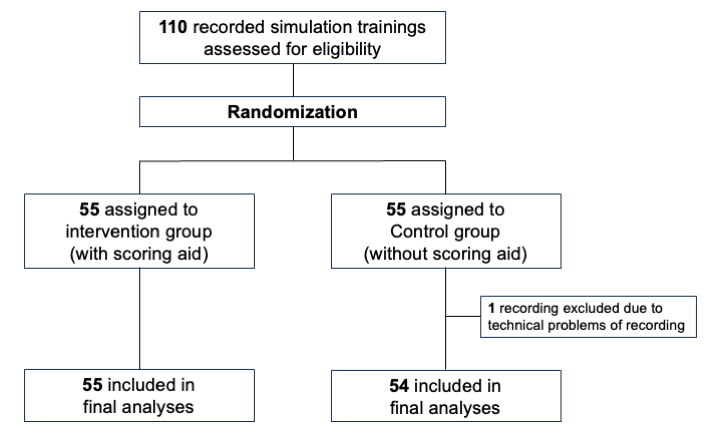

Supplement: Supplementary file 1 — Supplementary file1 Supplemental Figure 1. Flow chart (TIFF 915 KB) [file 415_2024_12825_MOESM1_ESM.tiff]
