# Supplementary material for: Effects of a scoring aid on glasgow coma score assessment and physicians’ comprehension: a simulator-based randomized clinical trial
Source: J Neurol. 2024 Dec 12;272(1):57. doi: 10.1007/s00415-024-12825-z (PMC11638317; doi:10.1007/s00415-024-12825-z)
Supplement: Supplementary file 2 — Supplementary file2 (DOCX 18 KB) [file 415_2024_12825_MOESM2_ESM.docx]

**Supplemental Table 1:** Participants’ demographics, baseline and performance characteristics, primary and secondary outcomes; n=109

| **Baseline and performance characteristics** | **n / median** | **% / IQR** |
| --- | --- | --- |
| **Demographics** |  |  |
| Age (years; median, IQR) | 32 | 30-34 |
| Female (n, %) | 66 | 60.6 |
| **Professional characteristics** |  |  |
| Stated to routinely use GCS in practice (n, %) | 84 | 77.1 |
| Physicians’ affiliations |  |  |
| Intensive care (n, %) | 28 | 25.7 |
| Emergency medicine (n, %) | 18 | 16.5 |
| Internal medicine (n, %) | 32 | 29.4 |
| Neurology (n, %) | 31 | 28.4 |
| Clinical experience (years; median, IQR) | 4 | 2.5-6 |
| Previous simulator training (n, %) | 51 | 46.8 |
| **Personal conditions prior to simulator training** |  |  |
| Working hours prior to participation (hours; median, IQR | 9 | 0-10 |
| Self-reported stress level hours prior to participation (rated from 0 to 10^a^; median, IQR) | 5 | 3-6 |
| **Overall performance characteristics** |  |  |
| GCS assessed (median, IQR) | 8 | 7-8 |
| Procedure of eye response assessed correctly (n, %) | 92 | 84.4 |
| Procedure of verbal response assessed correctly (n, %) | 82 | 75.2 |
| Procedure of motor response assessed correctly (n, %) | 61 | 56.0 |
| Number of commands directed to the patient during assessment (median, IQR) | 3 | 2-5 |
| Pain stimuli applied (median, IQR) | 1 | 0-1 |
| **Self-assessment of quality of GCS assessment** |  |  |
| Level of certainty that assessed GCS is correct (rated from 0 to 10^b^; median, IQR) | 8 | 6-9 |
| **Outcomes** | **n / median** | **% / IQR** |
| **Primary outcomes** |  |  |
| True GCS assessed correctly (n, %) | 57 | 52.3 |
| GCS assessment within a range of ±1 point around the true GCS of 8 (n, %) | 87 | 79.8 |
| Deviation from true GCS (median, IQR) | 0 | -1-0 |
| **Secondary outcomes** |  |  |
| Duration of GCS assessment  (seconds from first touch of the patient to calling a specific score; median, IQR) | 50 | 37-69 |
| **Understanding regarding assessment** |  |  |
| Checking eye response is recognized as the most challenging assessment (n, %) | 7 | 6.4 |
| Checking verbal response is recognized as the most challenging assessment (n, %) | 32 | 29.4 |
| Checking motor response is recognized as the most challenging assessment (n, %) | 70 | 64.2 |
| **Understanding regarding clinical applications** |  |  |
| Stated that patients with a GCS≤8 have to be intubated (n, %) | 28 | 25.7 |
| Stated that patients with a GCS≤8 do not have to be intubated (n, %) | 4 | 3.7 |
| Stated that intubation should be considered depending on the clinical context in patients with a GCS≤8 (n, %) | 77 | 70.6 |
| Maximal GCS attainable in intubated patients (median, IQR) | 11 | 9-11 |
| **Understanding regarding limitations** |  |  |
| Presumed interrater variability (rated from 0% to 100% certainty^c^; median, IQR) | 50% | 40%-70% |

_a_Predefined levels of stress: 0 representing no stress to 10 representing presumed maximal stress or highest stress level

_b_Predefined levels of certainty: 0 representing presumed complete uncertainty to 10 representing presumed absolute certainty

_c_Predefined level of certainty: 0% representing complete uncertainty and 100% representing absolute certainty that other physicians will assess a different GCS of the same patient with an identical clinical condition
